# At Last a Teaching University

**Published:** 1902-02-01

**Authors:** 


### AT LAST A TEACHING UNIVERSITY.

NEXT May the University of London will begin its real work as a teaching body. A suite of rooms in the buildings at South Kensington has been allocated for the purpose of teaching advanced physiology and experimental psychology, and establishing the necessary laboratories in connection with these subjects, and a commencement will be made by a course of lectures by Dr. Leonard Hill, F.R.S., on the Circulation. Dr. Waller, F.R.S., will also give a double course on (*a*) Signs of Life and (*b*) Animal Electricity. In October Professor Starling, F.R.S., will begin a course on the Sources of Animal Energies, and Dr. M. S. Pembrey a double course on (*a*) Heat and (*b*) Electricity. Rooms are also set apart for General Physiology and for Physiological Psychology, and a long list of eminent men forms the first panel of University lecturers. The chairman of the Board of Studies is Dr. Waller, F.R.S. Mr. Walter Palmer, M.P., has promised to contribute £2,000 for the purpose of providing scientific apparatus. May many others follow his example ! It is to be noted, however, that, admirable as is the proposal for the University to take physiology by the hand and to give such advanced courses as are now in contemplation, this is a very different affair and quite distinct from the ordinary daily grind of physiological teaching, suited to those anxious to obtain ordinary degrees, which ought to be given by a teaching university. This will have to be given at the proposed school of intermediate studies, wherever that may be. A proposal has recently been made that University College should be incorporated in the University of London, and that the land and buildings and endowments of the College should be placed at the complete disposal of the University ; and although this would, no doubt, be regarded with exceeding jealousy by some of the other medical schools, we cannot but see that if such a scheme could be set fairly afoot it would really provide exactly the nucleus of university teaching in the very centre of London which is wanted. If it should be possible to include anatomy and physiology among the subjects dealt with, the University of London would at once become a truly "teaching university" in regard not only to a large range of general studies, but also as to all the preliminary and intermediate subjects of medical education, the more technical and special studies, medicine, surgery, &c., being provided for in connection with the various hospitals. At a meeting of the Senate of the University of London held last week, it was formally announced that the Drapers' Company, believing that it would be for the good of higher education in London that the University College should be so incorporated with the London University, were willing to make themselves responsible for the debt on the College to the extent of £30,000, provided the bodies involved can arrange a scheme acceptable to the company before the end of September 1903. At the same meeting Sir Michael Foster, K.C.B., stated that he was authorised to announce that an anonymous donor was prepared to give to University

College £1,000 a year, redeemable either by himself or his executors by payment of £30,000, the conditions attached to the gift being the same as those attached to the offer of the Drapers' Company. So does money smooth the way for great enterprises, and so does wealth show that in good hands it is not ignoble.

